# EFFECT OF BASELINE VALUES ON INPATIENT REHABILITATION OUTCOMES AFTER TOTAL KNEE ARTHROPLASTY: A RETROSPECTIVE OBSERVATIONAL STUDY

**DOI:** 10.2340/jrm.v57.40443

**Published:** 2025-01-24

**Authors:** Martin MISSMANN, Michael J. FISCHER

**Affiliations:** 1 Austrian Workers’ Compensation Board AUVA, Innsbruck; 2 Ludwig Boltzmann Institute for Rehabilitation Research, Vienna; 3 Vamed Rehabilitation Center Kitzbühel, Kitzbühel, Austria; 4 Hannover Medical School MHH, Clinic for Rehabilitation Medicine, Hannover, Germany

**Keywords:** rehabilitation, total knee arthroplasty, outcomes, length of stay

## Abstract

**Objective:**

To compare inpatient rehabilitation outcomes after total knee arthroplasty (TKA) between groups with different baseline scores.

**Design:**

A retrospective observational study.

**Subjects:**

Patients with knee osteoarthritis who have previously undergone unilateral TKA.

**Methods:**

Patients participated in 3-week inpatient rehabilitation following TKA and were assessed for patient-reported outcome measures (PROMs), which included the Numeric Pain Rating Scale (NPRS), the Health Assessment Questionnaire (HAQ), the European Quality of Life 5 Dimensions 5 Level Version (EQ-5D-5L), and the Western Ontario and McMaster Universities Osteoarthritis Index (WOMAC). Furthermore, mobility scores for the range of motion (ROM) and the Timed Up and Go (TUG) test were recorded at the beginning and the end of rehabilitation. Patients were divided into quartile groups based on their initial examination scores.

**Results:**

329 patients were enrolled in the study. The study population consisted mostly of female patients (63.8% vs 36.2%) with a mean age of 68.25 (SD 9.24) years. The personalized 21-day in rehabilitation programme was safe for all patients and had no dropouts. Patients with better PROMs scores at T1 did not have the same potential for improvement in PROMs but showed effective improvement in mobility (η² = 0.103 for changes in the WOMAC vs η²=0.502 for changes in the TUG test).

**Conclusion:**

Regardless of the baseline scores, all patients presented significant improvements in both subjective and objective measures. Age and baseline PROMs or mobility scores did not have a significant effect on score development.

## LAY ABSTRACT

Osteoarthritis is a significant cause of pain and disability. Total knee arthroplasty (TKA) is a highly successful intervention for pain relief and function improvement in patients with end-stage osteoarthritis of the knee joint. After TKA, the patient’s movement is limited and restricted due to decreased muscle strength, pain, and swelling. Postoperative rehabilitation appears to be important for improving mobility and function. This retrospective observational study compared the outcomes of patients who began rehabilitation with good or bad mobility and those who rated their health condition as good or bad. Surprisingly it was found that all patients benefited from inpatient rehabilitation, regardless of their baseline values. However, other studies show that patients who do not receive inpatient rehabilitation also improve. The question arises as to whether all patients actually require the same level of rehabilitation to achieve their rehabilitation goals.

Osteoarthritis is a significant cause of pain and disability worldwide, affecting over 600 million individuals (1, 2). Premature joint degeneration can occur due to increased joint loading in obese individuals and unphysiological post-traumatic joint loading. Joint replacement surgery is in high demand among the elderly, who are most commonly affected by knee osteoarthritis (KOA) (3). Total knee arthroplasty (TKA) is a highly successful intervention for pain relief and functional improvement in patients with end-stage osteoarthritis (4). Short-stay protocols for TKA patients have emerged as a cost-effective solution to improve surgical techniques and perioperative care. Identifying several factors can decrease the length of stay (LOS) for inpatients after surgery and enable “fast track” or “rapid recovery” programmes (5).

After TKA, the patient’s movement is limited and restricted due to decreased muscle strength. In addition, range of motion (ROM) of the knee joint is reduced due to pain, haematoma, and swelling. Postoperative rehabilitation programmes appear to be important for improving function, restoring normal joint motion, and improving outcomes and mobility in patients after TKA (6). Intensive postoperative rehabilitation restores joint function, facilitates daily activities, and is crucial in preventing complications that may lead to readmission, as 30-day readmission rates in orthopaedics alone range from 2% to 14% (7). The improvement achieved during a standardized inpatient rehabilitation programme depends on the patient’s functional status on admission and the severity of their orthopaedic condition. Patients reporting low ability to cope with daily life and experiencing pain and orthopaedic dysfunction are likely to require a longer length of stay. Patients who show good progress at the start of their rehabilitation will reach their goals in a shorter length of stay.

The need for inpatient rehabilitation is not without controversy and depends on financial aspects that can be stressful for patients and may potentially affect joint replacement rehabilitation outcome (8, 9). The financial aspects are different in, for instance, Canada and the United Kingdom than in German-speaking countries, where most people have access to a public health system that covers the cost of 3 weeks of inpatient rehabilitation (10, 11). The success of rehabilitation needs to be monitored to justify the high cost of inpatient rehabilitation. In order to measure rehabilitation outcome, established measurement tools were used that measure both physical performance-based outcomes (PPO), and subjective assessments, known as patient-reported outcome measures (PROMs).

Given that all patients receive approximately the same multimodal therapy during a 3-week stay, the question arises as to whether all patients actually require the same level of rehabilitation to achieve the rehabilitation goals. For this reason, patients were divided into 2 groups based on their initial rehabilitation measurements. The main objective of this study was to compare rehabilitation outcomes between patients in the first and fourth quartiles of baseline values and to determine whether patients with different results require the same amount of rehabilitation. Secondary objectives of this study were to determine the effect of age on rehabilitation outcome, to describe relationships between PROMs and PPOs in their respective groups, and to identify the differences in behaviour between PROMs and PPOs results during the rehabilitation process.

## METHODS

### Trial design

This observational study was designed as a retrospective cohort study at Rehabilitation Center Kitzbühel, Austria. The Ethics Committee of the Medical University of Innsbruck (Ref: EC Nr:1158/2019) approved the study protocol, which was also registered with the German Clinical Trials Register (DRKS, registration number: DRKS00022854).

The study was conducted in accordance with Good Clinical Practice guidelines, the guiding principles of the Declaration of Helsinki, and was reported according to the Strengthening the Reporting of Observational Studies in Epidemiology (STROBE) Statement: guidelines for reporting observational studies (12).

### Participants

*Recruitment and eligibility criteria*. The study population consisted of consecutively recruited patients with lower extremity conditions who were referred to an inpatient orthopaedic rehabilitation facility.

*Inclusion criteria*. patients undergoing follow-up treatment after unilateral total knee arthroplasty (TKA) were included in this study. Mentally impaired patients or frail patients in need of intensive care were excluded from admission.

*Randomization and blinding*. A total of 329 patients, divided into groups based on their initial examination scores at baseline, were included in the cohort study. The groups were categorized as quartiles 1 (best results) and 4 (worst results). The therapists and patients were not informed of the group assignment of the participants.

### Interventions

All patients completed a 21-day interdisciplinary inpatient rehabilitation programme following TKA, in compliance with Austrian health regulations. During rehabilitation, patients underwent medical treatments for an average of 2–3 h per day. The total number of therapy minutes was at least 1,800. Patient-reported outcome questionnaires, both generic and disease-specific, were completed at the beginning of the rehabilitation process (T1) and at the end of the rehabilitation process (T2). Before completing the questionnaires, patients were informed about the use of their data for research and quality assurance purposes and signed a written informed consent. A rehabilitation team consisting of highly trained physicians, nurses, physical therapists (PTs), occupational therapists, and psychologists provided multimodal therapy. PTs selected exercises from a standardized catalogue recommended by the American College of Sports Medicine (ACSM) (13), considering individual surgical specifications and impairments. Physical therapy included active elements such as individual exercise, group exercise, and underwater exercise, as well as passive elements such as massage therapy, continuous passive motion (CPM), and electrotherapy.

### Outcome measures

Patients were referred to the rehabilitation facility from public health clinics and began their rehabilitation sequentially. Patient selection was based solely on medical reasons, without any financial considerations. All patients successfully completed the 21-day rehabilitation programme with no dropouts observed in the sample group. PPOs and PROMs were assessed at baseline (T1) and at the end of rehabilitation (T2).

### Physical performance-based outcomes (PPOs)

*Joint range of motion (ROM).* Several tools have been designed to measure joint range of motion (14). As in the present study, the universal full-circle goniometer was the preferred instrument for measuring active knee range of motion in the sagittal plane. For statistical evaluation, we converted the degree values to percentages of the accepted normal axial range of motion (15).

*Timed Up and Go (TUG) test.* The TUG test is used to assess a person’s mobility and measures the time needed to rise from a chair, walk 3 m, turn around 180°, walk back to the chair, and sit down while turning 180°. The TUG test is a highly reliable and valid method for quantifying functional mobility and is also a useful tool for tracking clinical changes over time (16).

### Patient-reported outcome measures (PROMs)

In order to ascertain information concerning the physical and mental health of our patients, as well as the impact of TKA on quality of life, a series of patient-reported outcome measures (PROMs) were employed. These included the HAQ, EQ-5D-5L, and WOMAC as generic and specific PROMs, and the NPRS for the assessment of pain. In the past, biomedical performance values were the primary endpoints in medical and health research. However, more recently, personal patient information concerning subjective well-being has also been incorporated into the assessment of treatment success (17, 18).

Terms such as quality of life (QOL), health-related quality of life (HRQOL), functional status, and well-being are often used interchangeably (Hossain), while in a more detailed sense, HRQOL is defined as a multidimensional concept, encompassing key domains as specified by the Centers for Disease Control and Prevention (CDC) (19).

*Numeric Pain Rating Scale (NPRS).* Pain has a major impact on physical, emotional, and cognitive functioning and is self-assessed by patients using rating scales such as the Numeric Pain Rating Scale (NPRS). The NPRS describes pain intensity on a scale of 0 to 10, where 0 is “no pain“ and 10 is “the worst pain imaginable“ (20).

*Health Assessment Questionnaire (HAQ).* The HAQ is one of the most widely used comprehensive, validated, patient-oriented outcome assessment instruments (21). It comprises 20 items with the following 8 categories: 1. Dressing and Grooming; 2. Arising; 3. Eating; 4. Walking; 5. Hygiene; 6. Reach; 7. Grip; 8. Common Daily Activities. For each category, patients reported the degree of difficulty they had in performing 2 or 3 specific subcategory items, with 4 possible responses for each item or component.

*European Quality of Life 5 Dimensions 5 Level Version (EQ-5D-5L)*. The EQ-5D-5L is a generic instrument that is used to measure 5 dimensions of health status, each of which consists of 5 levels: mobility, self-care, daily activities, pain/discomfort, and anxiety/depression (22).

*Western Ontario and McMaster Universities Osteoarthritis Index (WOMAC).* The WOMAC has been designed to measure 3 dimensions: pain, stiffness, and physical function (23). The WOMAC is considered the primary measure of efficacy for osteoarthritis trials and is a valid and reliable tool for the assessment of functional outcomes after TKA (24).

### Statistical methods

At the beginning of rehabilitation, no specific group assignment was made within the total sample of 329 participants, whereas the values of the descriptive statistics for the objective and subjective values were collected for the total sample, for female (*n* = 210) and male (*n* = 119) participants. Descriptive analyses were performed for pain (NPRS), generic PROMs (HAQ, EQ-5D-5L), specific PROMs (WOMAC), and performance measures (TUG and ROM) in either selection. The correlations between TUG and WOMAC scores were determined using Pearson’s correlation coefficient. Subsequently, the results for PROMs, ROM, and TUG were split into 4 quartiles to compare the best (quartile 1) and worst (quartile 4) performers. Significance was calculated using t-test, effect size using Cohen’s d, and partial eta squared to identify critical factors for rehabilitation success. Statistical significance was set at p < 0.05. All calculations were conducted with SPSS software version 21.0 (IBM Corp, Armonk, NY, USA).

## RESULTS

### Participants

**Fig. 1:**
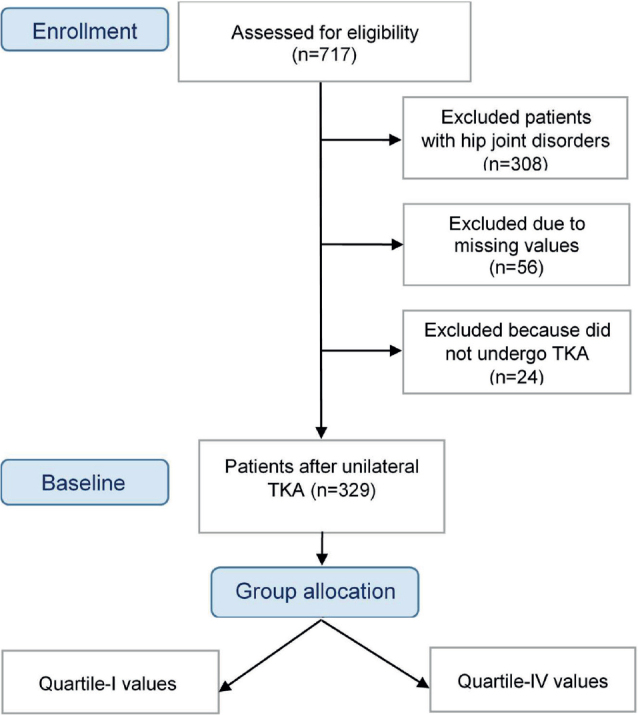
Flowchart of participants.

A total of 717 inpatients with lower limb conditions were initially assessed for eligibility. Of these, 308 were excluded due to hip joint disorders. Among the remaining 409 knee patients, data such as ROM values or TUG scores were missing for 56 patients. The study included 329 knee patients who had previously undergone TKA (Fig. 1). The main comorbidities observed were arterial hypertension (*n* = 58), diabetes mellitus (*n* = 15), substance abuse (*n* = 11), food and drug allergies (*n* = 3), and frailty (*n* = 3, including Parkinson’s disease, malignancy with living will, and tendency to fall). The study population consisted mostly of female patients (63.8% vs 36.2%) with a mean age of 68.25 (SD 9.24) years. The majority of patients were overweight (39.9%, *n* = 128) or obese (44.3%, BMI ≥ 25, *n* = 146), with only 16.7% (*n* = 55) having normal weight. Inpatient rehabilitation was completed without any complications. Additional information can be found in Table I.

**Table I:** Sociodemographic data and obesity classes at baseline

Factor	Total *n* = 329			Male *n* = 119			Female *n* = 210		
	Mean	Range	(SD)	Mean	Range	(SD)	Mean	Range	(SD)
Age	68.26	43–89	(9.24)	65.89	43–89	(9.4)	69.59	44–90	(8.9)
Height (cm)	167.05	144–196	(9.48)	176.27	161–196	(6.6)	160.62	144–181	(6.3)
Weight (kg)	83.23	46–133	(16.5)	93.28	65–133	(15.7)	77.53	46–127	(14.4)
BMI	29.75	19.4–46.3	(5.1)	29.99	24.8–45.3	(4.7)	29.62	19.4–46.3	(5.38)
	*n*	%		*n*	%		*n*	%	
Normal	55	16.7		15	12.6		40	19.0	
Overweight	128	39.9		50	42.0		78	37.1	
Obese	146	44.3		54	45.3		92	43.8	
Ob. class I	93	28.2		39	32.7		54	25.7	
Ob. class II	39	11.8		11	9.2		28	13.3	
Ob. class III	14	4.2		4	3.3		10	4.7	

SD: standard deviation; BMI: body mass index; obesity class I (BMI 30.0–34.9), II (BMI 35.0–39.9), III (BMI 40.0–49.9). Sex differences for age (height and weight): *p* < 0.001, for BMI *p* = 0.530.

### Treatment and changes in outcomes during rehabilitation

The participants underwent a 21-day inpatient rehabilitation programme, which included an average of 80 (10–215) min of doctor consultation, 287 (30–510) min of individual physiotherapy, 186 (30–390) min of massage therapy, and 178 (15–590) min of nursing care, including dressing changes. All patients successfully completed the rehabilitation programme without any dropouts observed. Only a minority of patients consulted a psychologist (*n* = 27) or dietologist (*n* = 102). In the total sample, all measures for ROM, TUG, HAQ, EQ-5D-5L, WOMAC, and pain (NPRS) showed significant score changes over the course of rehabilitation (*p* ≤ 0.001, see Table II), with a large effect size for ROM, medium effect sizes for TUG, NPRS, and WOMAC, and small effect sizes for HAQ and EQ 5D-5L values. Female patients showed significantly better improvement than male patients on the TUG test only (*p* = 0.01).

**Table II:** Knee joint mobility, Timed Up and Go test and Patient-Reported Outcome Measures at baseline (T1) and at the end (T2) of rehabilitation, temporal and gender differences in outcomes

	T1 mean	(SD)	T2 mean	(SD)	Δ mean	(SD)	T2-T1 *p*-value (Cohen’s d)	Male-Female *p*-value
ROM total	62.81	(12.10)	72.82	(8.09)	10.01	(7.19)	<0.001***	0.365
Male	64.36	(11.22)	73.89	(7.91)	9.53	(7.26)	(d=1.392)	
Female	61.93	(12.51)	74.06	(8.16)	10.28	(7.58)		
TUG total	12.45	(5.15)	9.61	(3.63)	-2.83	(2.82)	<0.001***	0.010**
Male	11.29	(4.78)	8.99	(3.92)	-2.31	(2.05)	(d=1.006)	
Female	13.10	(5.25)	9.66	(3.41)	-3.13	(3.13)		
HAQ total	0.38	(0.31)	0.27	(0.28)	-0.11	(0.24)	<0.001***	0.389
Male	0.31	(0.32)	0.21	(0.26)	-0.09	(0.24)	(d=0.460)	
Female	0.42	(0.30)	0.30	(0.29)	-0.12	(0.23)		
EQ5D total	1.77	(0.99)	1.55	(0.75)	-0.22	(0.92)	<0.001***	0.134
Male	1.67	(0.91)	1.50	(0.71)	-0.16	(0.83)	(d=-0.251)	
Female	1.83	(1.02)	1.58	(0.77)	-0.25	(0.96)		
NPRS total	4.27	(1.93)	3.03	(1.87)	-1.24	(1.77)	<0.001***	0.979
Male	3.98	(1.91)	2.74	(2.04)	-1.24	(1.75)	(d=0.700)	
Female	4.38	(1.93)	3.20	(1.75)	-1.24	(1.78)		
WOMAC total	75.88	(40.63)	49.65	(35.67)	-26.23	(30.51)	<0.001***	0.473
Male	73.86	(39.82)	46.02	(37.39)	-27.84	(30.10)	(d=0.860)	
Female	70.03	(41.13)	51.71	(34.57)	-25.32	(30.78)		

T1: beginning of rehabilitation; T2: end of rehabilitation; SD: standard deviation; Δ: delta, change of T2-T1 values; ROM: Range of Motion in percent of normal knee joint mobility; TUG: Timed Up and Go Test; HAQ: Health Assessment Questionnaire; EQ5D: European Quality of Life 5 Dimensions 5 Level Version; NPRS: Numeric Pain Rating Scale; WOMAC: score of the Western Ontario and McMaster Universities Osteoarthritis Index; level of significance

*** *p*<0.001;

** *p*<0.01;

* *p*<0.05

### Correlation of selected measures at the beginning and end of rehabilitation

Contrary to our initial expectations, we found a weak correlation between age and the other scores only for the TUG test at both T1 and T2. The BMI did not provide any meaningful prognostic information regarding mobility, pain, or WOMAC scores. Correlations between WOMAC scores at T1 and T2 with mobility (ROM and TUG) were weak or marginal. Moderate correlations were found between pain at T1 and WOMAC scores, as well as between pain at T2 and WOMAC scores at T2. In summary, we observed weak correlations between WOMAC and TUG, as well as pain at T1. Additionally, we found a weak correlation between pain at T2 and WOMAC at T2. Strong correlations were observed only for TUG at T1 with TUG at T2 and for ROM at T1 with ROM at T2, indicating similar patterns of behaviour (Table III).

**Table III:** Cross-tabulation of correlations of age and BMI at baseline and of ROM, TUG, WOMAC, and pain at baseline (T1) and at the end of rehabilitation

Factor	Age	BMI	ROM (T1)	ROM (T2)	TUG (T1)	TUG (T2)	WOMAC (T1)	WOMAC (T2)	NPRS (T1)	NPRS (T2)
Age	1	–0.24	0.03	–0.01	0.35	0.37	0.00	0.07	0.06	0.09
BMI		1	–0.11	–0.13	0.00	0.01	0.04	0.07	0.01	0.01
ROM (T1)			1	0.81	–0.30	–0.23	–0.31	–0.15	–0.20	–0.11
ROM (T2)				1	–0.25	–0.27	–0.30	–0.20	–0.22	–0.04
TUG (T1)					1	0.85	0.30	0.31	0.25	0.25
TUG (T2)						1	0.28	0.33	0.27	0.00
WOMAC (T1)							1	0.68	0.75**	0.54**
WOMAC (T2)								1	0.53	0.72
NPRS (T1)									1	0.56
NPRS (T2)										1

T1: beginning of rehabilitation; T2: end of rehabilitation; BMI: body mass index; ROM: range of motion in percentage of normal knee joint mobility; TUG: Timed Up and Go test; WOMAC: Western Ontario and McMaster Universities Osteoarthritis Index; NPRS: Numeric Pain Rating Scale; level of significance

*** *p* < 0.001;

** *p* < 0.01;

* *p* < 0.05.

### Effect of baseline score performance on rehabilitation outcome

At baseline, we chose the TUG as a mobility parameter and compared the PROMs and PPOs outcomes of the best (quartile 1) and worst (quartile 4) performers in the TUG. The difference between these 2 groups was highly significant for the change in ROM and TUG, significant for HAQ, and not significant for EQ5D, WOMAC, and pain (see Table IV).

**Table IV:** Score changes during rehabilitation for good and poor TUG, WOMAC, and overall performance at baseline

Admission: (Baseline, T1)	CHANGES (Δ) from admission to discharge (T2 –T1)											
	Δ ROM (SD)	part. η^2^	Δ TUG (SD)	part. η^2^	Δ HAQ (SD)	part. η^2^	Δ EQ5D (SD)	part. η^2^	Δ NPRS (SD)	part. η^2^	Δ WOMAC (SD)	part. η^2^
*TUG*												
Q1 (best) *n* = 102	7.4 (6.2)	0.59***	–0.9 (0.9)	0.48***	–0.06 (0.16)	0.12***	5.1 (10.3)	0.20***	–1.15 (1.37)	0.42***	–21.3 (24.3)	0.44***
Q4 (worst) *n* = 64	12.1 (8.0)	0.69***	–6.3 (3.8)	0.73***	–0.16 (0.33)	0.20***	5.9 (15.2)	0.13**	–1.59 (1.93)	0.41***	–26.1 (35.0)	0.36***
P (Q1:Q4)	<0.001***		<0.001***		0.007**		0.701		0.083(*)		0.301	
*WOMAC*												
Q1 (best) *n* = 85	8.1 (6.6)	0.61***	–1.9 (1.9)	0.50***	–0.06 (0.13)	0.16***	4.3 (11.0)	0.13***	–0.52 (1.68)	0.09**	–5.7 (17.0)	0.10**
Q4 (worst) *n* = 82	11.7 (7.6)	0.71***	–3.4 (3.2)	0.53***	–0.14 (0.29)	0.21***	8.7 (16.0)	0.23***	–1.67 (1.94)	0.43***	–48.1 (34.4)	0.66***
*P* (Q1:Q4)	0.001***		<0.001***		0.010*		0.041*		<.001***		<0.001***	
*Overall*												
Q1 (best) *n* = 83	6.6 (5.4)	0.60***	–1.4 (1.2)	0.58***	–0.03 (0.12)	0.051*	2.3 (7.6)	0.08**	–0.55 (1.13)	0.20***	–11.7 (18.3)	0.29***
Q4 (worst) *n* = 83	13.9 (8.0)	0.76***	–4.6 (3.8)	0.57***	–0.23 (0.30)	0.38***	10.2 (16.1)	0.29***	–1.96 (1.91)	0.52***	–43.2 (31.9)	0.65***
*P* (Q1:Q4)	<0.001***		<0.001***		<0.001***		<0.001***		<0.001***		<0.001***	

T1: beginning of rehabilitation; T2: end of rehabilitation; Δ: change T2–T1; SD: standard deviation in parentheses; Δ: delta, change of T2–T1 values; η^2^: eta-squared; Q1: quartile 1; Q4: quartile 4; ROM: range of motion in percentage of normal knee joint mobility; TUG: Timed Up and Go test; HAQ: Health Assessment Questionnaire; EQ5D: European Quality of Life 5 Dimensions 5 Level Version; NPRS: Numeric Pain Rating Scale; WOMAC: score of the Western Ontario and McMaster Universities Osteoarthritis Index; *p*: level of significance

*** *p*<0.001;

** *p*<0.01;

* *p*<0.05; Overall: sum of all scores.

**Fig. 2:**
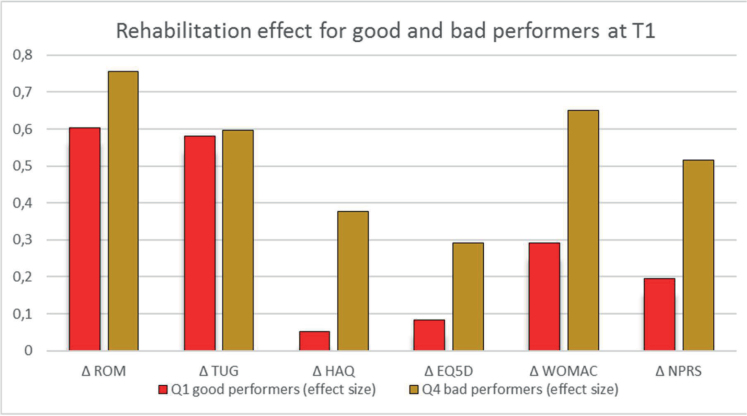
Effect of top and bottom scores at baseline on effect sizes for rehabilitation outcomes. Y-axis: Effect size of changes during rehabilitation in either group; T1: beginning of rehabilitation; Q1: quartile 1; Q4: quartile 4; Δ: change T2-T1; ROM: range of motion in percentage of normal knee joint mobility; TUG: Timed Up and Go test; HAQ: Health Assessment Questionnaire; EQ5D: European Quality of Life 5 Dimensions 5 Level Version; WOMAC: Western Ontario and McMaster Universities Osteoarthritis Index; NPRS: Numeric Pain Rating Scale.

Second, we selected the WOMAC at baseline as a specific PROM and compared the PROMs and PPOs outcomes between the best and worst performers in the WOMAC. The difference between these 2 groups was highly significant for the change in ROM, TUG, WOMAC, and pain, and significant for the HAQ and EQ5D. Third, an overall score was calculated at T1, combining PROMs and PPOs values. The analysis of the overall score showed a significant difference between the highest and lowest performing quartiles, as illustrated in Fig. 2. It is worth noting that quartile 4 patients exhibited greater improvement in all scores than quartile 1 patients, with comparable effect sizes for PPOs and larger effect sizes for PROMs.

## DISCUSSION

The aim of this study was to identify factors that predict rehabilitation potential in orthopaedic inpatient rehabilitation after TKA. The cost-effectiveness of joint replacement surgery is an issue from both a societal and a payer perspective, starting with the question of whether and to what technical level joint replacement should be performed (25). Depending on their preferences, physicians, therapists, and healthcare providers may focus on different aspects at the beginning of rehabilitation to create an efficient environment that provides optimal rehabilitation outcomes and patient satisfaction at minimum expense. For this reason, new rehabilitation methods are being established, such as in-home telerehabilitation via the Internet or home visit rehabilitation, which allows treatment without inpatient admission to a rehabilitation facility (26). Inpatient rehabilitation is uncommon in many countries and may not be necessary (27). In their review, Dávila Castrodad et al. found that inpatient rehabilitation did not provide better results than community or home-based therapy after TKA. For them, inpatient therapy did not provide any additional benefit (11).

### ROM outcome

Orthopaedic surgeons performing TKA have specific technical goals they expect to achieve at the time of surgery, including proper alignment, stability, and improved ROM (28). However, these technical parameters do not fully describe the actual condition of the patient after TKA. For example, they do not tell us how the patient feels and how the patient copes with the demands of daily life. In their study of the relationship between ROM and WOMAC scores after TKA, Miner and colleagues found a moderate correlation (R < 0.34) between poor knee mobility of less than 95 degrees of flexion and WOMAC scores 1 year after surgery. In contrast, WOMAC pain and physical function scores at 12 months correlated with both patient satisfaction and perceived improvement in quality of life, but knee flexion did not (29), partially contradicting our results.

In previous rehabilitation concepts, great efforts have been made to improve knee joint mobility after surgery, including the use of motorized devices such as continuous passive motion (CPM). In a meta-analysis, Yang et al. found that the use of CPM did not show a statistically significant improvement in postoperative ROM, except for medium-term passive knee extension ROM and long-term active knee flexion ROM. In addition, CPM therapy has no significant positive effect on functional outcomes (30). Their findings are consistent with our study, which showed a low correlation between ROM and WOMAC at any time point. Moreover, improvements in knee mobility during rehabilitation do not reliably indicate long-term mobility success, as Mutsuzaki et al. demonstrated in their study on joint range of motion after knee arthroplasty (31). The primary findings indicated that changes in the knee range of extension reached a plateau at 6 months post-TKA, while those in the knee range of flexion reached a plateau at 3 months post-TKA, which is typically later than the end of rehabilitation. We conclude that, within a certain range, assessment of knee joint mobility at baseline is not useful for predicting functional rehabilitation outcomes or patient satisfaction.

### TUG outcome

The TUG test is a recommended measure of function, balance, and walking ability and is one of the most commonly used performance-based outcome measures after TKA. While walking tests such as the 12-minute walking test (12MWT) or the 6MWT provide reproducible measures of effort tolerance in patients with chronic bronchitis, the TUG test is a recommended measure of function, balance, and walking ability and is one of the most commonly used performance-based outcome measures after TKA (32).

Its original purpose was to assess mobility and fall risk in elderly patients. Later, Kear et al. presented the findings of their study, which included normative reference values for individuals under the age of 60 years in the TUG. The results demonstrated a significant correlation with age, socioeconomic status, and health factors such as body mass index (BMI) and comorbidities (33). In addition, the study conducted by Williams and colleagues demonstrated that the TUG exhibited consistent results in children between the ages of 3 and 9, indicating good response stability (same-day retest) and test–retest reliability (34).

According to the results of a study by Givens et al., performance-based measures such as the TUG are more responsive than patient-reported outcome measures in the acute phase after TKA (35). Mizner et al. stated that patient perception fails to capture acute functional decline after TKA and may overstate long-term functional improvement with surgery (36).

They concluded that performance-based tests are needed to fully characterize changes in patients’ physical function after TKA that are not captured by patient-reported measures alone, which was confirmed in our study. Baseline TUG or ROM scores were mainly predictive of each other at the end of rehabilitation. Both ROM and TUG at baseline are not suitable measures for predicting perceived rehabilitation success because they provide limited information concerning the future behaviour of generic and specific PROMs.

### WOMAC outcome

Previous studies have found a discrepancy between WOMAC scores and actual mobility. Stratford et al. found that WOMAC does not fully reflect patient mobility (37). They pointed out that, in clinical practice, reliance on self-reported scores may lead to erroneous conclusions regarding patient mobility after TKA, which is consistent with our results. We concluded that the WOMAC total score did not fully predict rehabilitation outcomes in terms of mobility.

### Outcome and age

In general, poor rehabilitation outcomes after hip fracture are associated with the age of patients over 80 years (38). However, this was not the case in our study sample of 67 older patients. Here, in both quartile 1 (young patients aged 44 to 62 years) and quartile 4 (older patients aged 77 to 89 years), age was moderately correlated with TUG scores, but not with pain or WOMAC score. Authors such as Singh et al. mentioned the lack of a precise definition of “elderly patient”: They identified 20 criteria for “elderly”, of which only 3 related to chronological age, while in the other cases the term “age” was used as a surrogate for comorbidity (39). Alvis and Hughes listed typical changes in organs affected by age-related changes (40). Frailty is caused by a greater impairment of 1 of these systems or a combination of these impairments. (41). According to Rockwood’s Clinical Frailty Scale (FCS), the 3 frail patients in our sample scored 3–4 on the FCS. Although the patients in our study belonged to significantly different age groups, the difference in age-related effects was moderate. This contributes to the fact that patients with more severe impairments are usually cared for in nursing homes and not in rehabilitation facilities.

### Benefits of supervised rehabilitation

Our study confirms that positive changes in both PROMs and mobility scores are key factors contributing to the success of rehabilitation after TKA. All patients benefited from inpatient orthopaedic rehabilitation after TKA, regardless of sociodemographic factors or baseline scores of performance measures and PROMs. PROMs demonstrate significant self-perceived improvement during rehabilitation, particularly in patients with more pronounced complaints. The patients who reported fewer complaints at T1 did not have the same potential for improvement in the PROMs, but they did show an effective improvement in their mobility.

Inpatient rehabilitation after total knee arthroplasty (TKA) is associated with positive outcomes. However, this does not imply a necessity for inpatient rehabilitation. This issue has already been addressed by Chaudhry et al., who conducted a systematic review and meta-analysis which concluded that there is no significant benefit for supervised physiotherapy compared with unsupervised home exercise regimens for primary TKA patients. In their review, no significant long-term differences were found in any of the outcome measures, including both patient-reported outcome measures (PROMs) and range of motion (ROM) (42).

### Is inpatient rehabilitation after TKA actually useful for all patients?

We showed that the measures improved with rehabilitation in both patient groups, no matter whether the physical performance or the self-estimated values were at a higher or a lower level at the beginning of rehabilitation. This raises the question of whether there is any benefit to a 60-year-old patient improving their TUG from 7 s to 6 s, or, in another case, when the WOMAC score worsens from a good 20 points at baseline to 28 points.

Furthermore, healthcare stakeholders must consider whether they can and should provide comprehensive inpatient rehabilitation for all patients, particularly given the increasing current health expenditure (CHE) in an ageing society.

### Limitations of the study

This study demonstrated beneficial outcomes of inpatient rehabilitation after TKA. According to the local regulations, it was impossible to create a second sample without inpatient rehabilitation. However, it should be noted that patients with an increased frailty index were not admitted to this orthopaedic rehabilitation but were primarily cared for in a nursing facility or in a facility with a focus on geriatric rehabilitation.

A further limitation of the study was its retrospective design, which may have introduced statistical bias due to the potentially non-representative selection of the control group. However, this limitation is offset by the fact that all patients received equivalent treatment and that the 2 patient groups were recruited from the same collective. The 2 patient groups were not randomly formed; rather, they were constituted based on their respective outcome values.

It is recommended that future studies employ an RCT design that includes patients with and without inpatient rehabilitation following TKA, as well as results for follow-up at 6 and 12 months postoperatively. In Austria, the entitlement to postoperative rehabilitation and the structure of the treatments are explicitly defined. Consequently, such future randomized controlled trials must be multicentre studies that also include patients from outside Austria.

### Conclusion

All patients benefited from inpatient orthopaedic rehabilitation after TKA, provided that they were not frail. Older or younger age and high or low values in mobility measures or patient-reported outcome measures at the beginning of rehabilitation are not decisive for a beneficial score development during the course of rehabilitation. Future studies are needed to determine whether and to what extent inpatient rehabilitation is necessary after TKA.

## Data Availability

The datasets analysed in this study are not publicly available because of ethical and legal restrictions (data contain potentially identifying and sensitive patient information). If not already reported in this work, the authors may provide descriptive data on individual medical indicators for admission and discharge or the expected change due to inpatient healthcare for various groups and diagnoses. Requests for access to anonymized datasets should be directed to the corresponding author.
